# Changes in diving behaviour and habitat use of provisioned whale sharks: implications for management

**DOI:** 10.1038/s41598-020-73416-2

**Published:** 2020-10-12

**Authors:** Gonzalo Araujo, Jessica Labaja, Sally Snow, Charlie Huveneers, Alessandro Ponzo

**Affiliations:** 1 Large Marine Vertebrates Research Institute Philippines, Cagulada Compound, 6308 Jagna, Bohol Philippines; 2 Southern Shark Ecology Group, College of Science and Engineering, Flinders University, Adelaide, SA Australia

**Keywords:** Ocean sciences, Marine biology, Behavioural ecology

## Abstract

Whale shark (*Rhincodon typus*) tourism is increasingly popular at predictable aggregations around the world, but only a few use provisioning to ensure close interactions. Understanding the effects of provisioning on the behaviour of this endangered species is critical to manage this growing industry. We recorded the diving behaviour and habitat use of juvenile whale sharks (n = 4) for a mean of 49.5 provisioned and 33.8 non-provisioned days using temperature-depth-recorders. We found that time spent at the surface (< 2 m) between 6 am and 1 pm increased ~ sixfold, while timing of deep dives shifted from 4–10 am to 10 am–2 pm, i.e. near or at the end of the provisioning activities. The shift might be related to a need to thermoregulate following a prolonged period of time in warmer water. These changes could have fitness implications for individuals frequently visiting the provisioning site. Based on recorded amount of time spent in warm waters and published Q_10_ values for ectotherms, we estimate a 7.2 ± 3.7% (range 1.3–17.8%) higher metabolic rate when sharks frequent the provisioning site. The observed behavioural, habitat use, and potential fitness shifts should be considered when developing guidelines for sustainable tourism, particularly in light of new provisioning sites developing elsewhere.

## Introduction

Wildlife tourism is one of the fastest developing sectors and arguably the world’s largest tourism sector^1,2^. Tourism with sharks as the focal species is an increasingly popular activity, exceeding 590,000 tourists in 20 countries in 2012, and has likely doubled given a single mass tourism site in the Philippines now receives > 500,000 tourists annually^3,4^. Shark tourism developed rapidly over the past two decades^5,6^ and the socio-economic implications of this industry have been used to show positive attitude changes towards conservation by tourists^7^ and to advocate for conservation efforts due to the high non-consumptive value of sharks^8,9^. Shark tourism has, however, also been considered a threat to wildlife and ecosystems, with documented impacts including changes in physiology (e.g.^10,11^), seasonality, residency or abundance (e.g.^12–15^), space use (e.g.^16,17^), vertical activity (e.g.^17,18^), physical effects from divers (e.g.^19^), and overall dynamic body acceleration^20^.

The whale shark *Rhincodon typus* is the largest extant elasmobranch^21^ and a charismatic species that supports profitable tourism industries across global hotspots at which they aggregate^5,22–24^. Opportunities to snorkel or dive with whale sharks at these aggregations are numerous^21^, but only a few sites use provisioning to provide close interactions between tourists and whale sharks (i.e. Oslob in the Philippines; Cenderawasih Bay, Gorontalo, Triton Bay and East Kalimantan in Indonesia;^25^). Previous studies at Oslob have shown that provisioning activities doubled the residency times of whale sharks and increased the probability of resighting over time^15,26^, increased human-shark-boat contact^27^, and affected local reef ecosystem^28^. However, the fitness implications of these changes have not yet been quantified.

Tagging studies can help elucidate some of the knowledge gaps in the behavioural ecology of the species. Tagging of juvenile whale sharks suggest that they are a primarily epipelagic, staying above 240 m most of their time, but known to reach depths of over 1900 m^29,30^. Such epipelagic fish are known to move frequently through the water column, which has been suggested to be driven by the need for prey detection, reduced energy expenditure, thermoregulation, or navigation^31–34^. In planktivorous elasmobranchs, diving behaviour has been closely associated with the diel movements of zooplankton, highlighting the use of such behaviour to find prey^29,35^. Thermoregulation in whale sharks has also been suggested when whale sharks dive to temperatures below 25 °C^36^, which sharks might preferentially select to slow down metabolism following a period of constant feeding^30^.

**Figure 1:**
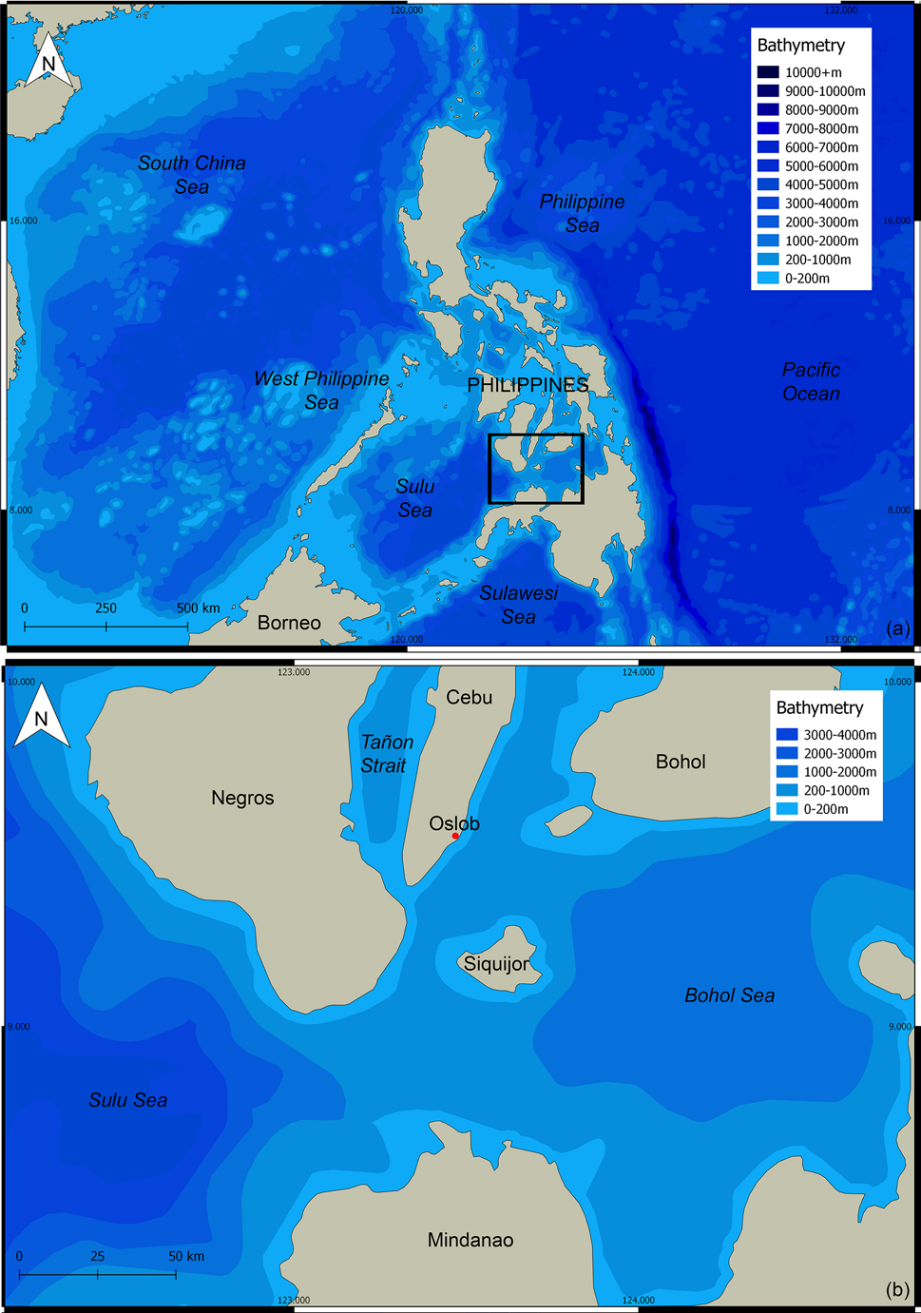
Map of the Philippines (**a**) and a larger version of the rectangle to the study site of Oslob in southern Cebu (**b**), indicated by a red dot.

Here, we closely examine the habitat use and diving behaviour of four whale sharks at a provisioned aggregation in Oslob (Cebu, Philippines; Fig. 1) using temperature-depth-recorder tags. The occasional absence from the study site allowed insight into these patterns when sharks were away, as confirmed through daily photo-identification. We compared the depths and water temperatures frequented by whale sharks on days during which they visited the provisioning site *vs.* days during which they did not visit the provisioning site. As temperature is a key ecological abiotic factor that directly affects physiological processes^37,38^, we then used the water temperature differences during provisioning *vs.* non-provisioning days to estimate changes in metabolic rate.

## Results

**Figure 2:**
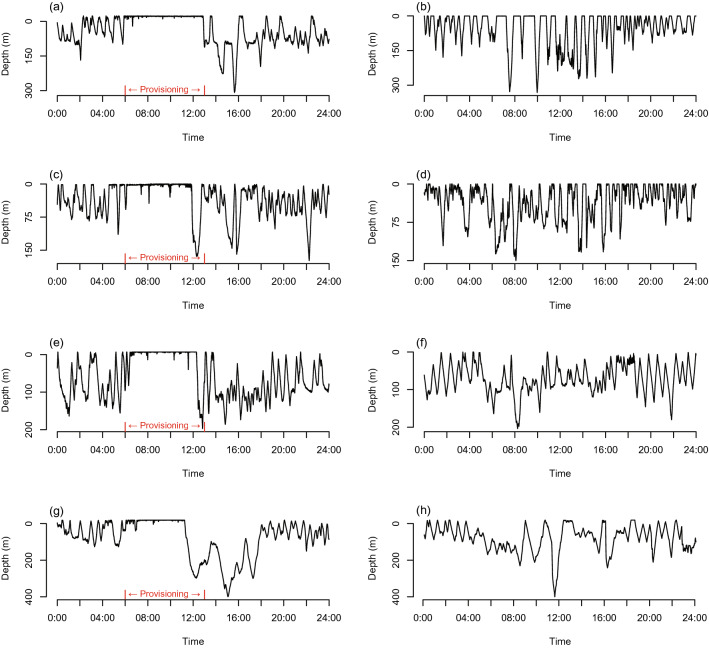
A randomly selected dive profile from a provisioned (left column) and non-provisioned (right column) day for shark P-385 (**a**,**b**), P-432 (**c**,**d**), P-403 (**e**,**f**) and P-480 (**g**,**h**). Note the daily provisioning period identified in red text.

**Figure 3:**
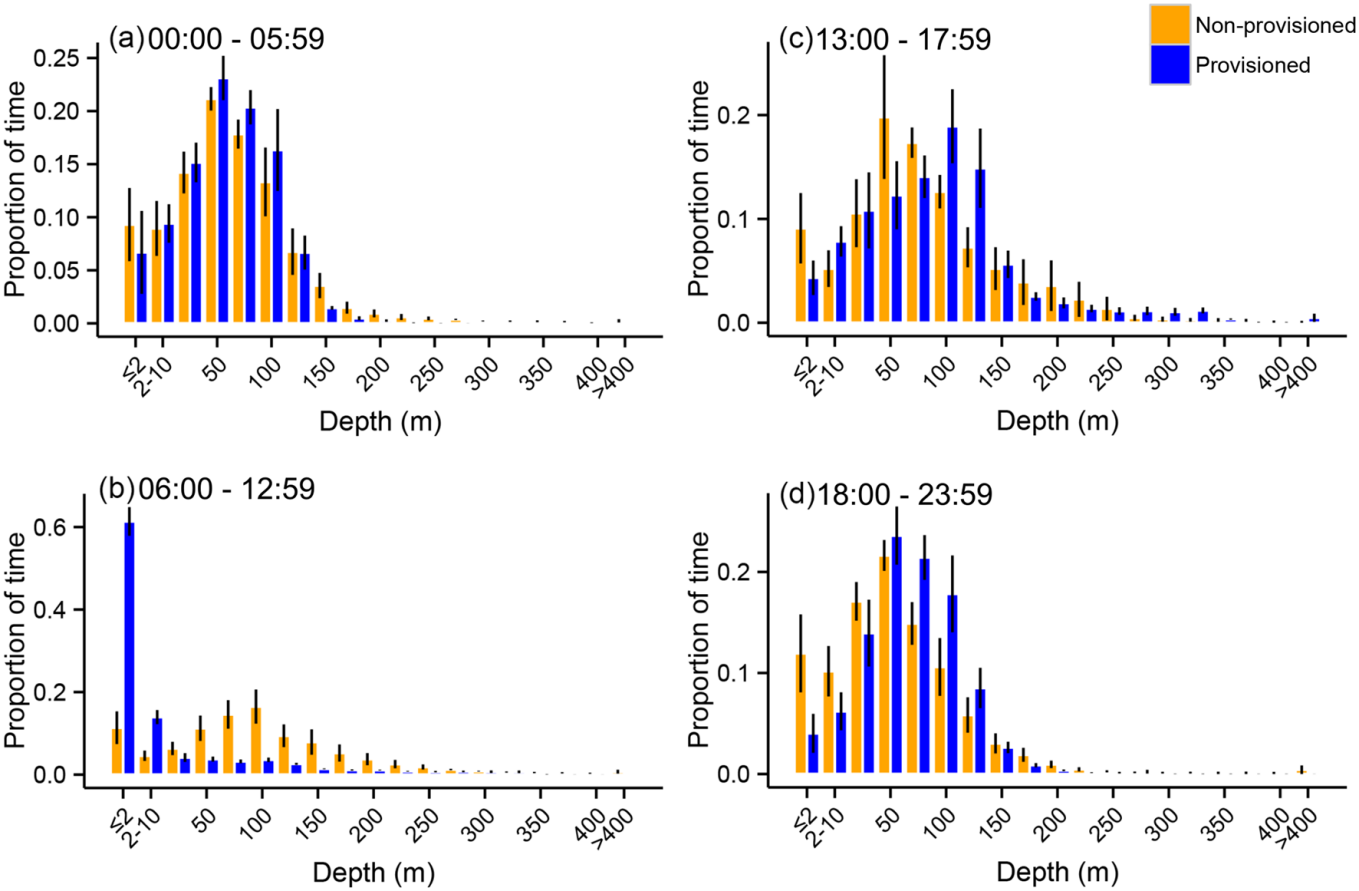
Binned depth use averaged for the four focal sharks during the early morning (**a**), provisioning (**b**), afternoon (**c**) and evening (**d**) periods. Error bars are ± 1 SE. Contrast represents provisioned (blue) and non-provisioned (orange) days.

**Figure 4:**
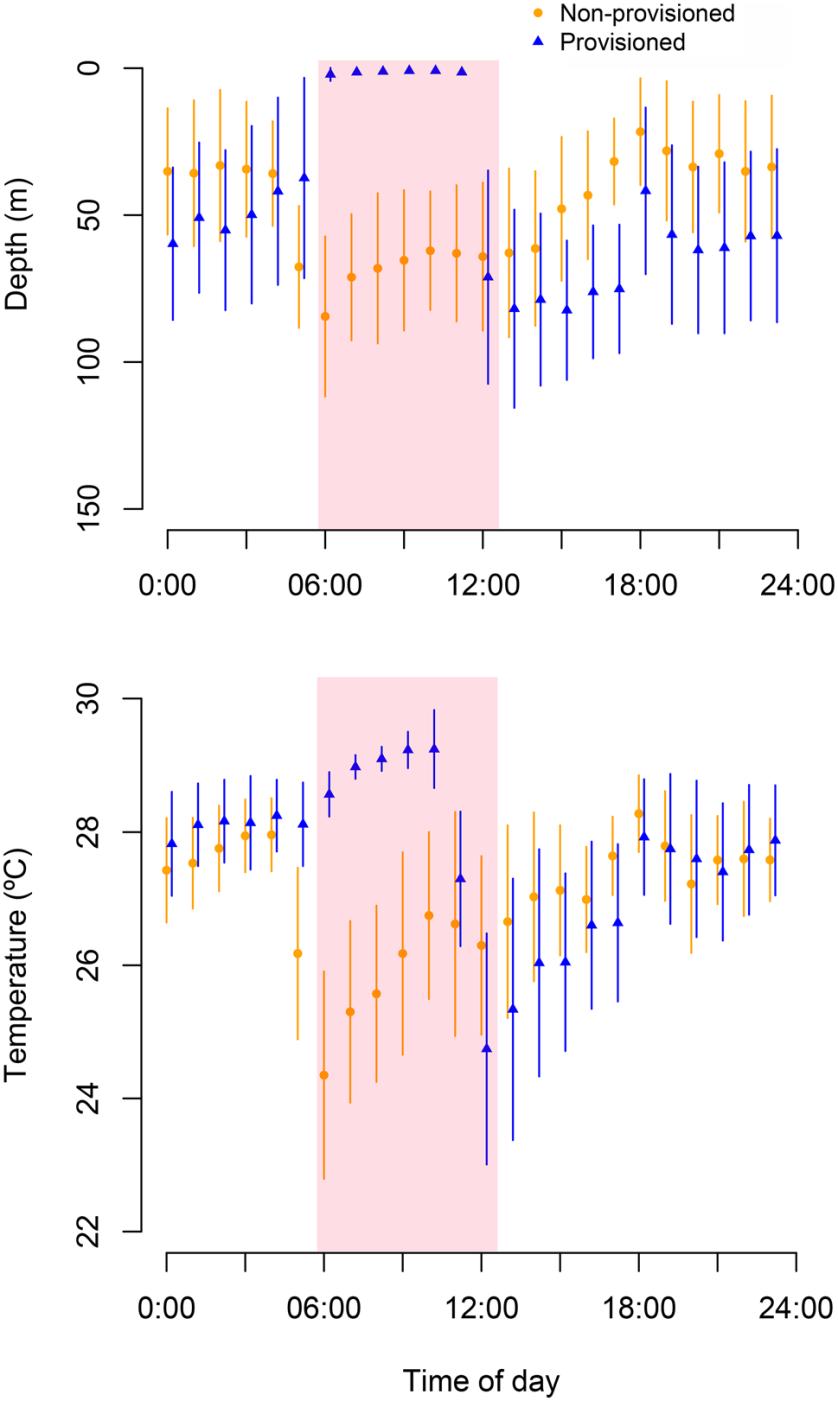
Median depth (**a**) and temperature (**b**) use for all sharks during provisioned (triangles) and non-provisioned (dots) days. The shaded area represents the provisioning period.

**Figure 5:**
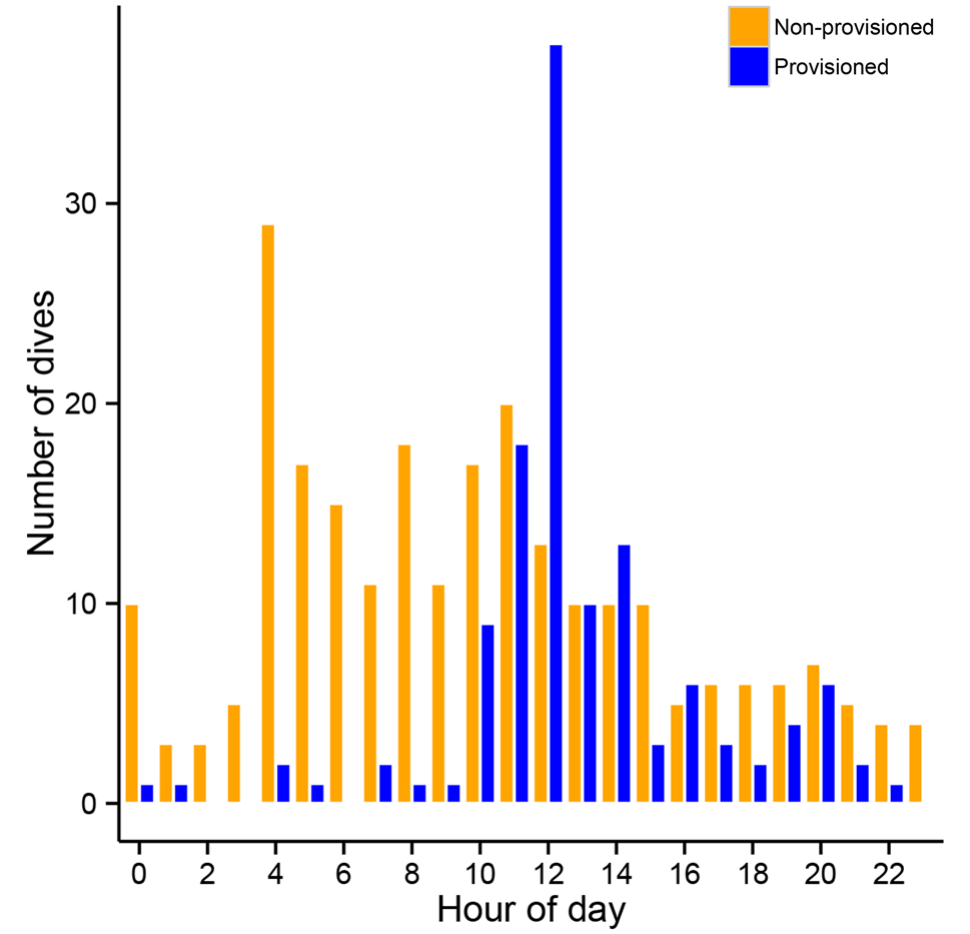
Temporal distribution of deep dives (> 200 m) performed by whale sharks on provisioned and non-provisioned days. Contrast represents provisioned (blue) and non-provisioned (orange) days.

**Figure 6:**
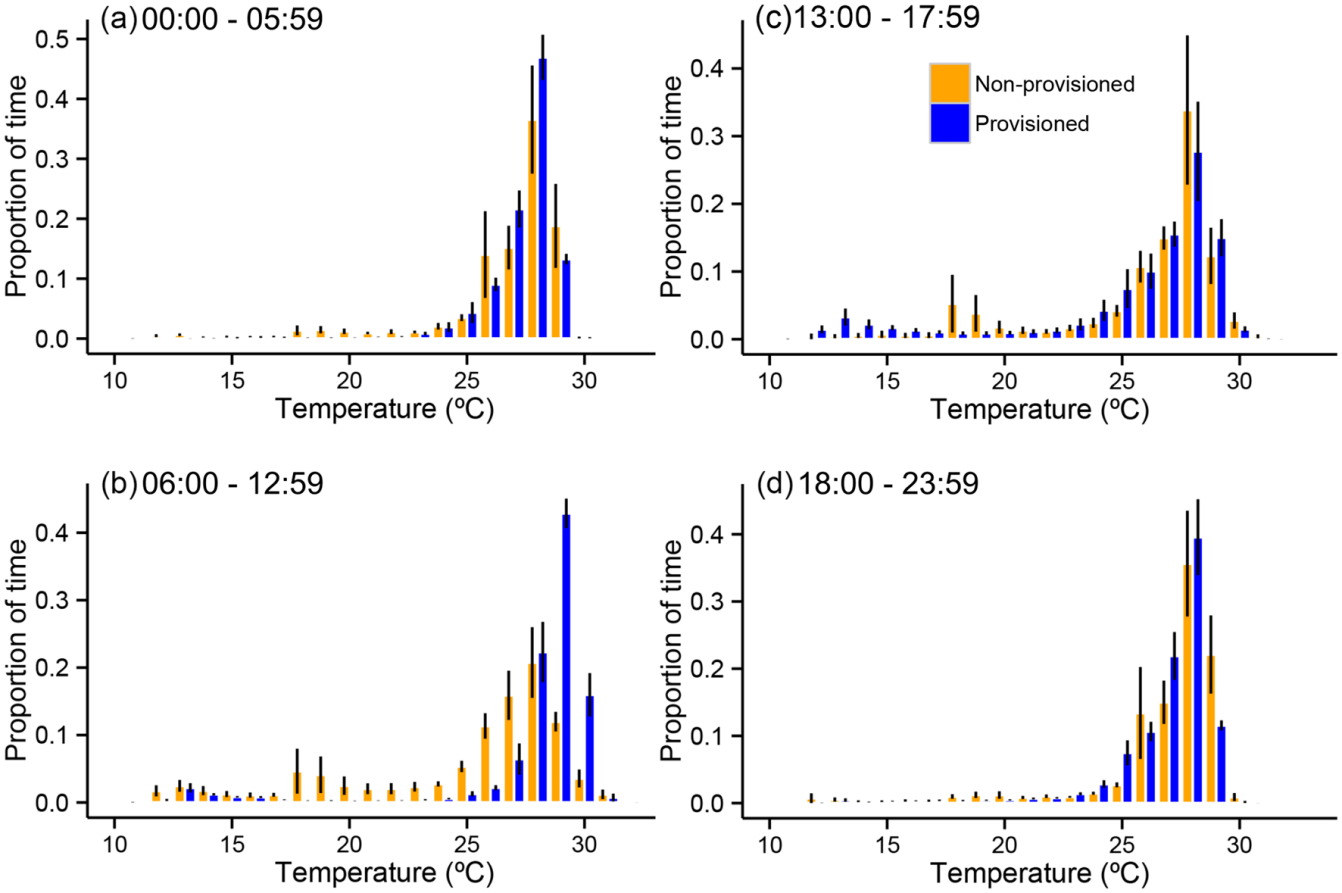
Binned temperature use averaged for the four focal sharks during the early morning (**a**), provisioning (**b**), afternoon (**c**) and evening (**d**) periods. Error bars are ± 1 SE. Contrast represents provisioned (blue) and non-provisioned (orange) days.

Diving data were collected for an average of 49.5 (± 20.5 standard error [SE]) provisioned days and 33.8 (± 10.8) non-provisioned days for each of the four focal sharks (Table 1). The depth and temperature use patterns of these sharks differed markedly between provisioned and non-provisioned days (Figs. 2, 3, 4, 5, 6), described in the following section.

**Table 1:** Information for four whale sharks tagged with temperature-depth-recorder tags in Oslob, Cebu, Philippines.

Shark-ID	Sex	Size (m)	Date first tagged	No. of days sighted before deployment	No. of days tagged	Provisioned days
P-385	F	5.5	09-Jul-13	224	103	42%
P-403	M	6.5	03-Jun-13	289	138	79%
P-432	M	4.5	13-Jul-14	719	66	42%
P-480	M	5	08-Jun-13	304	26	69%

### Depth

The proportion of time averaged across all sharks revealed sharks spent 10.5% of their time at the surface (< 2 m) between 6 am and 1 pm on days when they did not visit the provisioning site (Figs. 2, 3). In contrast, sharks spent 57.6% of their time at the surface during this same time period on days they were provisioned. This is supported by significant differences in the overall median depth as explored through linear mixed effects models (F_7,4961_ = 166.4, *P* < 0.001). A summary of depth use is presented in Table 2.

**Table 2:** Summary of depth data for tagged whale sharks during provisioned and non-provisioned days.

Shark ID		Depth (m)			
	Contrast	Mean	SD	Min	Max
P-385	Provisioned	58.59	64.89	< 2.0	515.37
	Non-provisioned	60.47	69.1	< 2.0	771.37
P-432	Provisioned	37.17	48.22	< 2.0	339.87
	Non-provisioned	53.02	49.31	< 2.0	385.37
P-403	Provisioned	60.52	60.77	< 2.0	657.37
	Non-provisioned	73.31	76.14	< 2.0	737.87
P-480	Provisioned	54.96	53.64	< 2.0	346.37
	Non-provisioned	95.39	71.73	< 2.0	416.37

Planned contrasts revealed that median depths were much shallower during the provisioning period (coefficient = −5.20, SE = 0.25, z = −21.10, *P* < 0.001) and deeper during the afternoon (coefficient = 1.49, SE = 0.22, z = 6.65, *P* < 0.001) and evening (coefficient = 1.42, SE = 0.18, z = 7.94, *P* < 0.001), on provisioned compared to non-provisioned days (Fig. 4). Median depths during the early morning prior to provisioning time were not significantly different between provisioned and non-provisioned days (coefficient = 0.33, SE = 0.19, z = 1.79, *P* = 0.23; Table 3).

**Table 3:** Effects of provisioning on median temperature and depth use, explored through planned contrasts.

Time of day	Depth	Temperature
Early morning (00:00–05:59)	z = 1.79, *P* = 0.23	z = −3.53, *P* = 0.002
Provisioning period (06:00–12:59)	z = −21.10, *P* < 0.001	z = −11.94, *P* < 0.001
Afternoon (13:00–17:59)	z = 6.65, *P* < 0.001	z = 4.24, *P* < 0.001
Evening (18:00–23:59)	z = 7.94, *P* < 0.001	z = 0.77, *P* = 0.85

The distribution of deep dives (> 200 m) differed significantly between provisioned and non-provisioned days (χ^2^ = 230.1, df = 23, *P* < 0.001; Fig. 5). On provisioned days, sharks primarily performed deep dives near or just after the end of the provisioning period (10 am–2 pm), with a particularly high frequency of deep dives occurring from 12 noon to 1 pm (Figs. 2, 5). In contrast, on non-provisioned days, sharks dove beyond 200 m relatively consistently between 4 and 10 am and infrequently at other times of day (Fig. 5).

### Temperature

Linear mixed effects models revealed that provisioning had a significant effect on the overall daily median water temperature experienced by whale sharks (F_7,4961_ = 53.2, *P* < 0.001; Figs. 4, 6). Planned contrasts revealed that median temperature was much higher during the provisioning period (coefficient = 0.49, SE = 0.04, z = 11.94, *P* < 0.001) and lower during the afternoon (coefficient = −0.18, SE = 0.04, z = −4.24, *P* < 0.001) on provisioned compared to non-provisioned days. Median temperature during the evening was not significantly different (coefficient = −0.02, SE = 0.03, z = −0.77, *P* = 0.85) on provisioned compared to non-provisioned days (Table 3). Median temperature during the early morning (0–6 am) was higher between provisioned and non-provisioned days (coefficient = 0.11, SE = 0.03, z = 3.53, *P* = 0.001). A summary of temperature use is presented in Table 4.

**Table 4:** Summary of temperature data for tagged whale sharks during provisioned and non-provisioned days.

Shark ID		Temperature (ºC)			
	Contrast	Mean	SD	Min	Max
P-385	Provisioned	27.05	3.73	12.52	31.75
	Non-provisioned	26.27	3.97	11.16	32.14
P-432	Provisioned	27.66	2.85	12.67	31.59
	Non-provisioned	27.49	3.48	12.62	31.84
P-403	Provisioned	27.2	3.39	12.38	32.12
	Non-provisioned	26.77	3.79	12.28	30.81
P-480	Provisioned	27.74	3.20	13.08	31.25
	Non-provisioned	25.65	3.82	17.64	31.72

#### Metabolic rate

**Figure 7:**
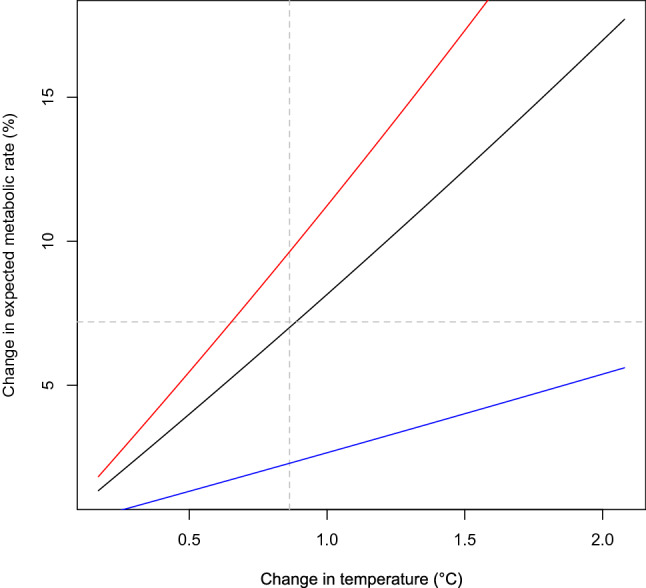
Changes in expected metabolic rate based on a Q_10_ of 2.19 (black), within a range of 1.3 (blue) to 2.9 (red), the extremes of published metabolic Q_10_ values for ectotherms. The dashed lines represent the change in temperature of + 0.9 °C and the expected metabolic rate estimated in this study (7.2%), based on a Q_10_ of 2.19.

Differences in surrounding water temperatures between the provisioning and non-provisioning periods (2.83 ± 0.45 °C) led to an overall difference in water temperature between provisioned and non-provisioned days of 0.86 ± 0.43 °C (range 0.17–2.08 °C). Based on a Q_10_ of 2.19 (the mean Q_10_ of published metabolic studies for ectothermic taxa), expected metabolic rate increased by 7.2 ± 3.7% (range 1.3–17.8%). The change in expected metabolic rate was sensitive to Q_10_, with the average metabolic rate increase ranging 2.3 to 10.0% for a Q_10_ of 1.3 to 2.9, respectively (Fig. 7), the extremes of published metabolic Q_10_ values for ectotherms.

## Discussion

Understanding the impacts of provisioning activities on whale sharks is challenging given the long-lived, slow growing, and wide-ranging nature of the species. Here, we show that provisioning elicited a pronounced shift in depth and temperature use in resident whale sharks visiting Oslob. While this behavioural shift cannot be generalised across all individuals sighted in Oslob, the study shows that sharks regularly frequenting the site are affected by provisioning. In the four tagged sharks, provisioning resulted in whale sharks spending *ca*. six times as much time at the surface during provisioning hours (6 am–1 pm) compared to non-provisioning days. Morning provisioning was followed by different shark depth-use patterns throughout the remainder of the day: the timing of deep dives shifted, with few dives exceeding 200 m occurring during the early morning (when they were primarily performed in the absence of provisioning) and most occurring at the end of the provisioning period. This change in depth use also led to whale sharks being exposed to warmer temperature during provisioning hours and overall during provisioning days, which affected metabolic rate estimates based on the amount of time spent at different water temperatures.

Whale sharks dive up to 1928 m^30^ and recent evidence shows that demersal zooplankton are significant prey for the species^39^. The highly predictable food provided in Oslob at the water surface is a major deviation from natural foraging conditions for this species, which typically capitalise on vertically migrating prey that move shallower during the night^40^ and otherwise display a yo-yo type diving behaviour the remainder of the time (i.e. moving up and down the water column; e.g.^30,41^; this study, non-provisioned days). Thus, while the difference in depth use in response to daily provisioning is not surprising, the magnitude of the behavioural shift is considerable and raises important considerations for this endangered species.

The whale sharks at Oslob that feed at the surface for extended periods of time (often 6 am to 1 pm) are exposed to higher temperatures and more direct sun exposure than would be typical (e.g.^30,42–45^). The prolonged sun exposure on shallow waters with a sandy substrate reflects sunlight which might cause skin darkening on the ventral side of the animals (GA, pers. obs.). It is unclear whether this has any impact on the animals. The propensity to perform deep dives after feeding at the provisioning site may be a response to the need to thermoregulate following a prolonged period of surface feeding in tropical waters, which at times exceeded 31 °C. As ectothermic fish, whale sharks might move vertically through the water column to regulate physiological processes after spending time in warm or cool water^36^. Diel patterns in depth use have previously been hypothesised to relate to either heat dissipation or post-feeding thermotaxis to improve digestive uptake^30,36,46^.

The physiological or fitness impacts of high temperatures on sharks are not well understood. A study investigating the effects of increased temperatures (4 °C) on juvenile epaulette sharks *Hemiscyllium ocellatum* showed significantly decreased growth rates and the ability to regulate their thermal environment through movement^47^. The higher water temperature during provisioning hours, i.e. ~ 2.5 °C, and amount of times spent at the provisioning site was sufficient to result in a metabolic rate increase of ~ 7%. A recent study has shown some thermal inertia in the body temperature of the whale shark due to its large body size, which might reduce the overall metabolic cost estimated here^48^. The study, however, also confirmed that the body temperature of whale shark is affected by ambient temperature, i.e. body temperature decreased by ~ 8 °C to a minimum of 19 °C after spending 4 h at ~ 400 m ^48^. Body temperature is also more likely affected by ambient temperature when filter-feeding, due to the large volume of water passing through the gills^36^. Since the tagged whale sharks spent most of the provisioning hours (~ 7 h) feeding at the provisioning site, and that filter-feeding is the predominant behaviour while at the site^27^, whale shark body temperature (and therefore metabolic rate) is likely to increase as a result of spending time at the provisioning site, regardless of the thermal inertia of this large-bodied species.

The need to slow down metabolism following prolonged feeding at high water temperatures might explain deep diving to cool waters following the provisioning hours at the surface^30^. The observed yo-yo diving behaviour may contribute to reducing energy expenditure, as deep-diving behaviour has been shown to be less energetically costly than horizonal swimming in several shark species including in white^49^ and whale sharks^41,50^. The energy burden from the increased metabolic rate might also be compensated for by the provisioning, with whale sharks being provisioned ~ 350 kg/day of food in Oslob. Whilst it is possible that whale sharks consume enough food to counter the increased metabolic expense during their daily presence at the site, it is worth noting that this could in itself create an ecological trap, particularly in an oligotrophic environment^10^. Bioenergetic models (e.g.^11^) are necessary to accurately assess the effect of whale shark provisioning in Oslob, including changes across seasons (i.e. with more or less food naturally available locally away from the provisioning site).

The scaling relationship between metabolic rate and body mass is an on-going debate (e.g.^51,52^) and small errors can result in large differences in metabolic rate estimates when extrapolating to megafauna. For this reason, we did not attempt to measure absolute metabolic rate, but instead reported the relative difference in metabolic rates based on exposure to different temperatures. We also acknowledge that the unknown Q_10_ value for whale sharks hinders our ability to infer strong ecological implications. The use of the mean Q_10_ across ectotherm taxa and extreme Q_10_ values to gauge sensitivity of metabolic rate showcase that the observed change in temperature is sufficient to result in an increased metabolic rate, possibly up to ~ 10%. We also acknowledge that metabolism is not directly influenced by external temperature but body temperature, and that the large body size of whale sharks might lead to some thermal inertia^48^.

Available technology did not permit measurement of body temperatures using internal sensors, because this would have required restraint of the subject animals, which was not possible owing to logistical constraints. However, the assumption that the external temperatures measured by the sensors provided a measure of body temperature of the sharks is reasonable, given that whale sharks are ectotherms. Such use of external temperature as an indicator of body temperature has also been used in previous studies (e.g.^36,41^). More research on the effects of water temperature on the metabolic rate of whale sharks is needed to accurately quantify how wildlife tourism might affect whale shark energetics, particularly in cases like Oslob where shifts in temperature use are observed. The large size of the species might make this logistically difficult, but equipment such as the mega-flume^53^ and whale sharks held in captivity could contribute towards making this possible.

Changes in diving behaviour and habitat use may have ecosystem-wide consequences. Whale sharks and their associated fauna likely play a role in the cycling of nutrients vertically between the meso- and bathypelagic zones with the epipelagic zone, and horizontally across vast distances through which they move^21,45,54,55^. Mobile predators, like grey reef sharks *Carcharhinus amblyrhynchos*, have been estimated to egest ~ 95 kg of nitrogen daily onto reef ecosystems at remote atolls in the Pacific^56^. Changes to movement and habitat use patterns of sharks could alter how these ecological roles are played. Energy and nutrient transfer, including microbiomes at the individual or species level^57^, across habitats as facilitated by key species such as the whale shark, are essential for ecosystem processes and biodiversity^54^. Indeed, deep-dwelling air breathing species like toothed whales and birds can cycle iron from great depths to the surface and land^58^. Altering such natural processes can have wider implications than initially thought and should merit closer examination when determining the sustainable use of an endangered species, particularly with a key player of ecosystem processes such as the whale shark.

A limitation of using TDR tags is their lack of geographical reference. However, understanding the immediate habitats from where the animal was tagged, and where the tag was retrieved, can give an idea of the general area used. The Bohol Sea reaches a minimum temperature of 11.6 °C with a constant thermocline running between 200 and 1800 m from 14 to 11.6 °C respectively^59^. Whale sharks leaving the provisioning site appear to dive deep immediately after provisioning stops. They can access the cool thermocline of the Bohol Sea at a relatively close distance to Tan-awan (< 1 km, > 200 m). Interestingly, the deep dives rarely exceeded 12 °C, suggesting they likely stayed within the Bohol Sea, and probably within relative proximity to the provisioning site (Supplementary Fig. 1). On one occasion, individual P-385 dove to 583 m at 10.8 °C within 24 h from being present at Tan-awan. This is cooler than the Bohol Sea’s lowest temperature, possibly indicating the animal travelled to the Sulu Sea, adjacent to the west, which reaches temperatures of 9.9 °C. Gordon et al.^59^ coincidentally shows temperatures of the Sulu Sea of 10.8 °C at a depth of 500–600 m. These deep dives could have an exploratory purpose for both prey and/or location (see^34^), or predatory avoidance as suggested for other species (e.g. leatherback turtles^60^) although not many whale shark predators are generally present in the general vicinity to the provisioning site (e.g. tiger or white shark *Carcharodon carcharias*^61^). A vessel collision or large propeller strike could potentially trigger a similar response.

The present study is limited by the inability to have a true control situation, and we acknowledge the lack of previous baseline data for the whale sharks tagged, wherein an ideal study we would have collected data before provisioning activities took place. Obtaining such control data is often impossible in the case of wildlife tourism studies as it is extremely rare that scientists have the opportunity to collect data at a tourism site prior to the tourism activity starting. This is typically due to these sites only becoming known following tourism development. As a result, previous studies have used data from other sites as ‘pseudo-controls’ because a control situation at the same site was not available (e.g.^13,62^). In Oslob, feeding occurs daily and it was not possible to request non-provisioning days to collect data at the site when feeding was not occurring. Even if we were able to do so, many sharks frequenting Oslob (including the four sharks included in our study) have changed their behaviour at that site^27^ and might therefore still show unnatural feeding behaviour at the site if provisioning was not occurring. Instead, we opted to compare habitat use and metabolic rate of whale sharks on provisioned vs. non-provisioned days. In addition, we deployed the same tags on seven whale sharks at a different location (Panaon Island, Southern Leyte, *ca* 220 km east of Oslob; see^63^) to further determine the ‘normal’ temperature and depth use of whale sharks in the region. Unfortunately, only two TDRs were successfully recovered with only one still functioning, further highlighting the difficulty of working with this species and the hurdles of a ‘control’ study. This shark showed no differences in depth and temperature use when compared to non-provisioned days of Oslob whale sharks (Supplementary Fig. 2).

## Conclusions and management implications

In Oslob, whale sharks are provisioned off of small paddle boats and use suction-feeding while staying vertically in the water, with this behaviour increasing with increased residency^27^. Although vertical feeding occurs naturally, the predictability of this behaviour for a sustained number of hours is unique^27^. Based on the long residency periods of some sharks^15^ and the major shift in depth use and water temperature leading to an increase in the expected metabolic rate presented here, it is clear that provisioning alters the behaviour of the whale sharks frequently visiting Oslob. Provisioning of elasmobranchs continues to be a debated topic, with the general understanding that a lack of baseline data on the biology, ecology, and physiology of these species complicates the interpretation of findings from provisioning sites^6,64,65^. However, indication that such activities might have detrimental effects to the physiology and ecology of endangered species should prompt management to follow the precautionary principle. This is particularly relevant for whale sharks which are listed as Endangered under the IUCN Red List of Threatened Species^66^, in Appendix II of the Convention on the International Trade of Endangered Species (CITES, 2003), in Appendices I & II of the Convention on Migratory Species of the United Nations (CMS, 2017), and for which the Concerted Actions for the Whale Shark (UNEP/CMS/CA12.7, 2017) recommended careful examination of tourism interactions with the species. In light of this and with four provisioning sites now developed in Indonesia at Cenderawasih Bay in West Papua^67^, Gorontalo in Sulawesi (Himawan, pers. comm.), Triton Bay^25^, and East Kalimantan (Authors, pers. obs.), and other sites being developed in the Philippines (Authors, pers. obs.), legislation and regulation is necessary to limit the impacts of provisioning on this mobile and endangered species.

The present study provides evidence of the effects of wildlife tourism on whale sharks and potential implications for their metabolic rate and habitat use, and ultimately improves our understanding of behavioural responses to anthropogenic influences. Further research is needed to quantify the effects of provisioning on the energy budget of whale sharks and to understand any long-term behavioural effects on this long-lived species. Such information will enable managers to account for the potential effects of wildlife tourism on the energy balance, fitness and ultimately population viability of this globally threatened species.

## Methods

### Ethics statement

This study was carried out in accordance with the ethical guidelines and approval, and in collaboration with the Department of Agriculture-Bureau of Fisheries and Aquatic Resources of the Republic of the Philippines, under whose management the whale shark falls. No animal was constrained and the methods employed were minimally-invasive in nature. The work was authorised by the Municipality of Oslob, duly represented at the time of the study by Hon. Mayor R. Guaren.

### Study site

The Municipality of Oslob is situated *c.* 125 km south of Cebu City, Cebu Province in the Visayas region of the Philippines (Fig. 1). Whale shark provisioning activities commenced in late 2011 at Barangay Tan-Awan (9° 27′ 46" N, 123° 22′ 48" E) and have been regulated since Jan 7, 2012 by a local ordinance (revised in Apr 2012). Whale sharks are provisioned ~ 350 kg of sergestid shrimps daily from 6 am to 1 pm from small outrigger boats at the surface. Daily photo-identification (henceforth photo-ID) of individuals began on Mar 31, 2012 and has been ongoing since. The Bohol Sea reaches ~ 2000 m depth and minimum temperatures of ~ 12 °C, whereas the Sulu Sea adjacent to the West is deeper and cooler, reaching 4400 m and ~ 10 °C^59^. At least three whale shark aggregations within the Bohol and Sulu Seas, with a degree of connectivity between them, have been documented^15,45,63^.

### Tagging

In Oslob, four sharks were tagged with Cefas G5 temperature-depth-recorder tags (Cefas Technology Limited, Suffolk, UK; https://www.cefastechnology.co.uk; henceforth TDRs) between Jul 2013 and Jul 2014 (Table 1) using a Hawaiian-sling spear pole to deliver a titanium anchor 10 cm into the subdermal layer. TDRs were programmed to record depth (± 0.33 m) and temperature (± 0.1 °C) every 5 s with the exception of two deployments, during which depth was sampled every 30 s. During the study period, each tagged whale shark was absent infrequently, but sometimes for extended (i.e., days to weeks), periods of time from the provisioning site. Daily photo-ID at the provisioned site was used for each shark to record their presence (henceforth a ‘provisioned day’) *vs.* days when tagged sharks were not sighted (henceforth a ‘non-provisioned day’). Although it is possible that whale sharks visited the site and were not photo-identified by researchers, it is unlikely given that the four tagged whale sharks feed from the provisioned food when present^27^ and at least three 1-h surveys are conducted daily. Shark size (m) was visually estimated as described in Araujo et al.^15^. Raw data were visually examined in 24-h blocks to assess and correct for sensor drift during a deployment.

### Data analyses

Depth and temperature were analysed by calculating the median and inter-quartile range (IQR) of recorded depths and temperatures for every hour of every day for each shark. Medians were used because of strong skew in the distribution of depth and temperature in some hourly time blocks. We then used linear mixed-effect models with planned contrasts to test the hypothesis that attendance at the provisioning site affects shark depth use and temperature throughout the day. Specifically, we created an eight-level treatment variable based on four time periods corresponding to early morning (00:00–05:59), the provisioning period (06:00–12:59), afternoon (13:00–17:59), and evening (18:00–23:59), and two provisioning levels (i.e., provisioned or non-provisioned). We then used planned contrasts to compare shark depth and temperature use within each time block between provisioned and non-provisioned days. Planned contrasts between the pairs of time blocks were used to test the difference between provisioned vs. non-provisioned days, instead of comparing all combinations of time blocks which would have unnecessarily reduced the power of the test^68^. We nested date within shark ID as random effects to account for repeated measures. Response variables were square-root transformed prior to analysis to reduce heteroscedasticity. Model assumptions were checked using diagnostic plots, and the *acf* function to test for auto-correlation in the linear mixed-effects models following Zuur et al.^69^. All statistical analyses were performed using R 4.0.0 GUI 1.71^70^.

Whale sharks occasionally perform extended, deep dives exceeding 200 m in depth (e.g.^30^). To determine whether provisioning influenced the frequency and timing of these dives, we compared the temporal distributions of dives > 200 m performed on provisioned and non-provisioned days using a Chi-square (χ^2^) test.

#### Metabolic rate

Thermal sensitivity varies substantially across ectotherms and is not well established for elasmobranchs^71^. Since no Q_10_ value is currently available for whale sharks, we used the mean Q_10_ across ectotherm taxa (2.19;^72^). We also estimated the rate of metabolic rate change based on the two most extreme, recent, and reliable Q_10_ values (1.3–2.9)^73,74^. The use of these two extreme values enabled us to account for the uncertainty in Q_10_ estimates and to assess the sensitivity of metabolic rates to changes in Q_10_.

Changes in metabolic rate were estimated using the recorded difference in water temperature and published Q_10_ and the following equation (based on the Q_10_ formula and solved for the percentage metabolic rate change):

$$ {\text{Metabolic rate change}}\; \left( \% \right) = \left( {Q10^{{\frac{\Delta t}{{10}}}} - 1} \right) \times 100 $$

where Δt is the difference in average water temperature encountered by whale sharks between provisioned and non-provisioned days, and Q_10_ is the thermal sensitivity of metabolic rate.

## Supplementary information

Supplementary information

## Data Availability

All identification data is hosted on the online database ‘Wildbook for Whale Sharks’ (www.whaleshark.org). Tag data will be made freely available upon manuscript publication.
