# Supplementary material for: Changes in diving behaviour and habitat use of provisioned whale sharks: implications for management
Source: Sci Rep. 2020 Oct 12;10:16951. doi: 10.1038/s41598-020-73416-2 (PMC7550605; doi:10.1038/s41598-020-73416-2)
Supplement: Supplementary file 1 — Supplementary information [file 41598_2020_73416_MOESM1_ESM.docx]

**Supplementary Material**

**Changes in diving behaviour and habitat use of provisioned whale sharks: implications for management**

Gonzalo Araujo^1*^, Jessica Labaja^1^, Sally Snow^1^, Charlie Huveneers^2^, Alessandro Ponzo^1^

^1^Large Marine Vertebrates Research Institute Philippines, Cagulada Compound, Jagna, 6308, Bohol, Philippines

^2^Southern Shark Ecology Group, College of Science and Engineering, Flinders University, Adelaide, SA, Australia

*Correspondence: Gonzalo Araujo, g.araujo@lamave.org | +639054043833


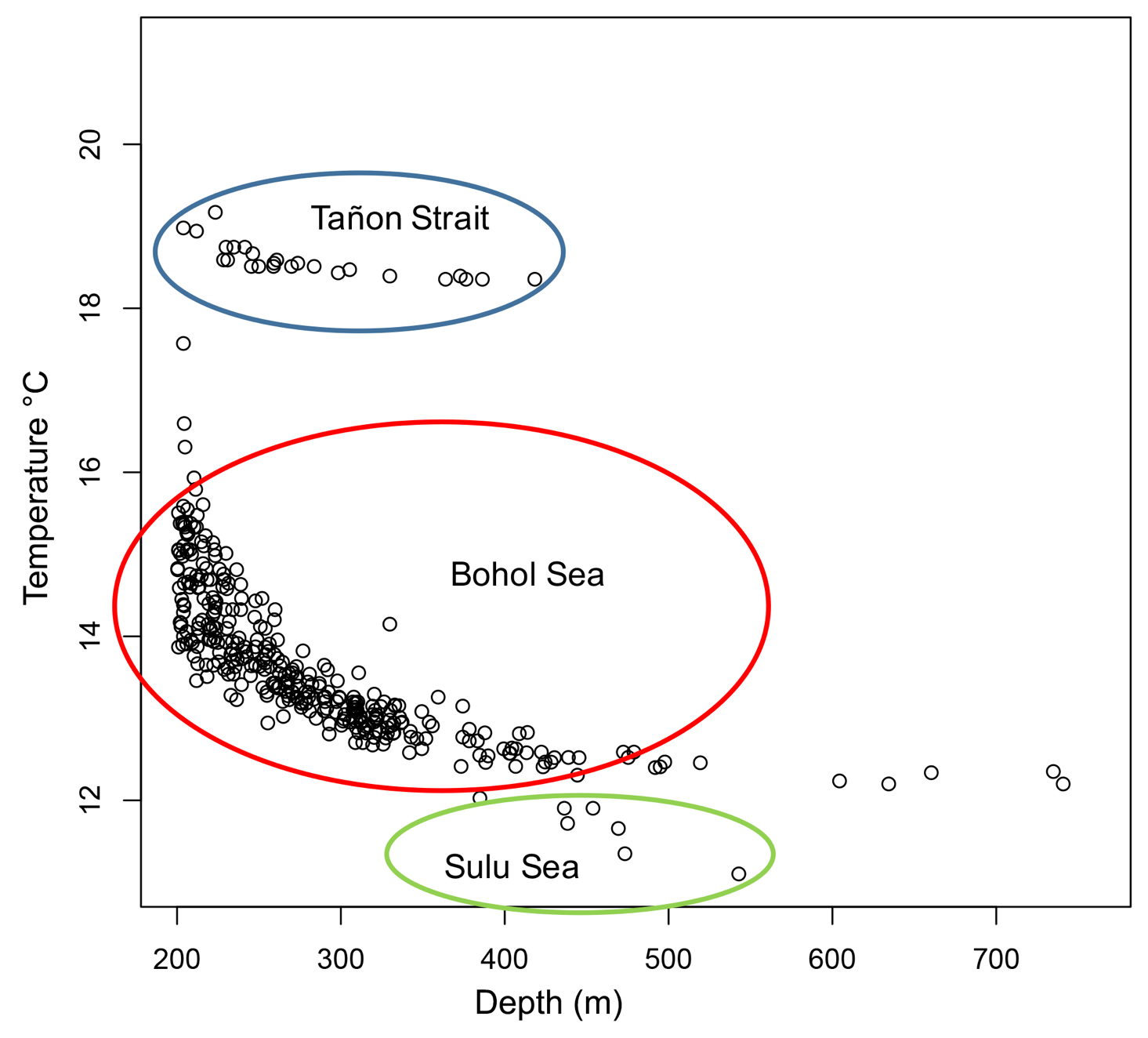


**Supplementary** **Fig. 1.** Temperature readings at deep dives (>200 m; *n* = 369) for all four sharks. Dives are grouped into probable geographic locations of deep dives, based on minimum temperatures for the Tañon Strait (blue), Bohol (red) and Sulu Seas (green), from Gordon et al. [1] and Hayasaka et al. [2].


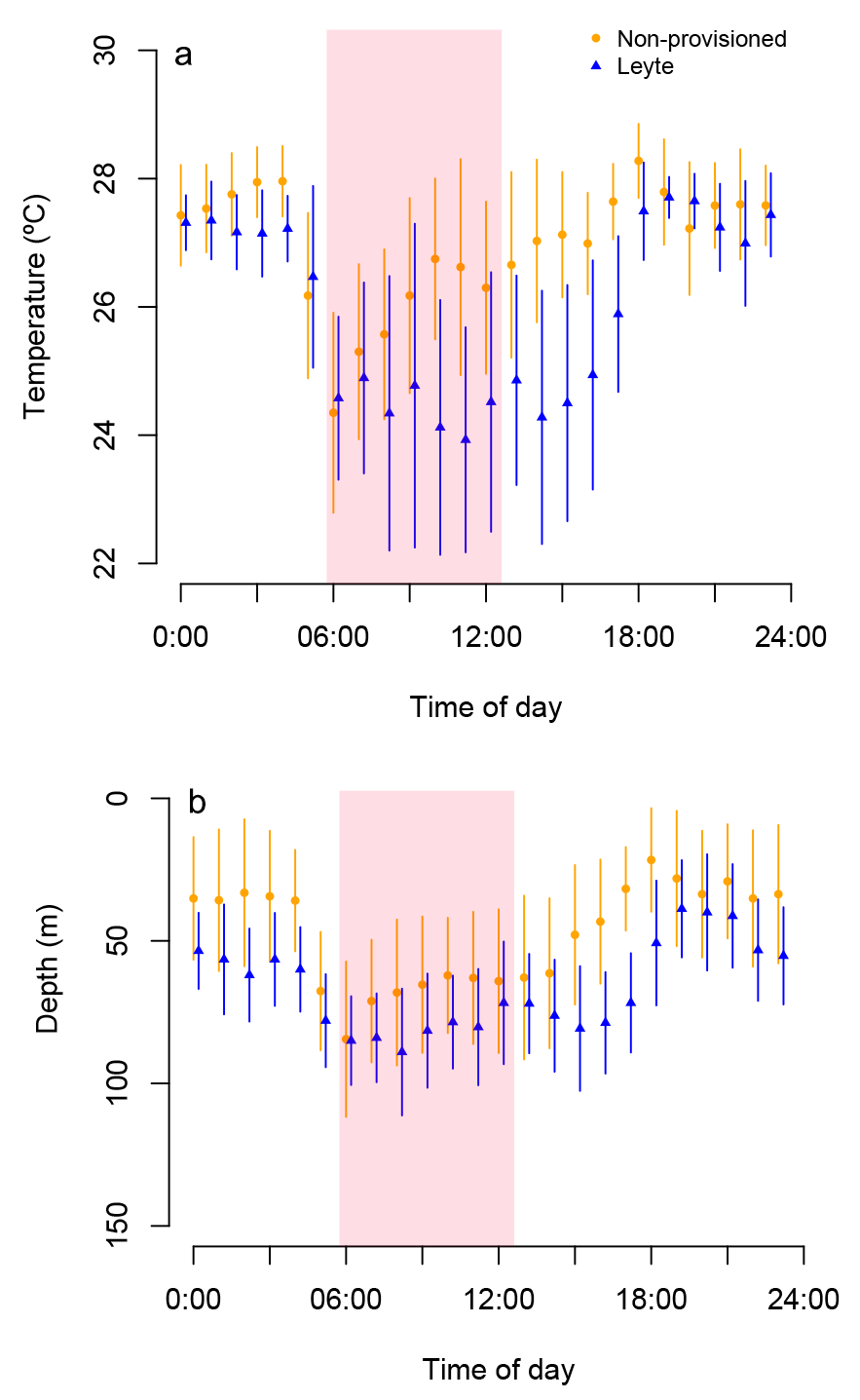


**Supplementary** **Fig. 2.** Temperature (a) and depth (b) use comparison between one shark (P-507) tagged at Panaon Island, Southern Leyte, and sharks tagged at Oslob during non-provisioned days.

**References**

1. Gordon, A. L., Sprintall, J., & Ffield, A. Regional oceanography of the Philippine Archipelago. *Oceanography* **24(1)**:14-27 (2011).
2. Hayasaka, S., Oki, K., Tanabe, K., Saisho, T., & Shinomiya, K., 1987. On the habitat of *Nautilus pompilius* in Tanon Strait (Philippines) and the Fiji Islands. *In*: Saunders, W. B., Landman, N.H., Nautilus: the Biology and Paleobiology of a Living Fossil. (Plenum Press, New York, **pp. 179–200**, 1987).
